# Effects of Indica Black Rice, Konjac Powder and Water on the Quality Characteristics of Gluten-Free Taichung Sen 17 Rice Bread

**DOI:** 10.3390/foods14223831

**Published:** 2025-11-08

**Authors:** Jin-Kuan Jiang, Shin-Yu Chen, Chih-Wei Yang, Hui-Shan Shen, Sheng-Dun Lin

**Affiliations:** 1 Department of Food Science and Technology, Hungkuang University, 1018, Sec. 6, Taiwan Boulevard, Shalu District, Taichung 433304, Taiwan; a5880561z@gmail.com (J.-K.J.); n91768oris@sunrise.hk.edu.tw (C.-W.Y.); roxenshen33@gmail.com (H.-S.S.); 2 Department of Food Science, National Pingtung University of Science and Technology, Pingtung 912301, Taiwan; sychen@mail.npust.edu.tw

**Keywords:** gluten-free, rice flour, rice bread, konjac glucomannan, texture, physicochemical quality, bioactive component, antioxidant

## Abstract

Rice is a safe and widely consumed gluten-free grain; however, breads prepared from white rice flour, such as Taichung Sen 17 (TS17), are prone to rapid staling and typically lack sufficient dietary fiber and bioactive compounds. To address these limitations, this study investigated the effects of partially replacing TS17 flour with indica black rice (B) flour, varying water content, and incorporating the natural hydrocolloid konjac glucomannan (K) on the quality of rice batter and bread. Compared with TS17 flour, B flour contained higher levels of total dietary fiber, total phenols, total anthocyanins, and antioxidant capacity. Substituting 15% of TS17 with B flour slightly increased the bread volume from 1032 mL to 1042 mL under 92% water addition (1.01-fold). More notably, it significantly increased the volume from 872 mL to 917 mL under 107% water (1.05-fold), and from 642 mL to 775 mL under 122% water (1.21-fold). However, higher substitution levels of B flour resulted in a reduction in loaf volume. Further incorporation of 2% K under 122% water conditions (TS17B15K2.0-122) resulted in the highest loaf volume of 1063 mL, representing a significant 1.37-fold increase compared to TS17B15-122 (775 mL), and exhibited the slowest staling rate after 24 h. Although K significantly improved bread texture and moisture retention, excessive addition may accelerate staling due to insufficient water availability in the formulation. These findings highlight that combining TS17 flour, B flour, and konjac gum represents a feasible and sustainable strategy for developing functional gluten-free baked products targeted at health-conscious and gluten-intolerant consumers.

## 1. Introduction

Wheat bread is one of the most widely consumed foods worldwide due to its desirable appearance, flavor, and texture, and it serves as a staple in human diets. Gluten, the major protein in wheat flour, plays a critical role in conferring viscoelastic and cohesive properties to dough, thereby enabling gas retention and structural development during breadmaking [1]. In the absence of gluten, carbon dioxide retention during yeast fermentation is markedly reduced, resulting in bread with coarse and crumbly texture, reduced loaf volume and specific volume, inferior mouthfeel, and shortened shelf life [2]. Moreover, gluten can trigger adverse immune responses in susceptible individuals, necessitating the complete avoidance of gluten-containing foods. For patients with gluten-related disorders, a strict gluten-free diet remains the only effective treatment [3,4]. Consequently, producing gluten-free bread with quality comparable to conventional wheat bread remains a considerable technological challenge [5,6,7].

Rice, one of the world’s most important staple crops, has been recognized as a safe cereal for individuals with wheat allergy or celiac disease due to its distinct protein composition [8,9]. This makes rice an attractive gluten-free raw material for bread formulations, either used alone or in combination with other gluten-free starches. Gluten-free rice breads are typically produced using white rice flour alone or blended with starches such as corn, potato, or tapioca. White rice is particularly favored for its low cost, mild flavor, and low allergenicity [10]. However, rice flour alone cannot fully replicate the functional role of wheat flour in breadmaking, as it lacks the structural components required to form a viscoelastic dough [11]. When used as a partial replacement, rice flour can be incorporated up to 30% without severely compromising product quality.

Rice is also a dietary staple in Taiwan, yet the successful development of bread made entirely from rice flour remains limited. To investigate the influence of rice flour properties on bread quality, our laboratory previously prepared four types of rice flours with amylose contents ranging from 9.2% to 30.5% via air-jet milling. Results demonstrated a positive correlation between amylose content and bread volume, with rice bread produced from Taichung Sen 17 (TS17, *Oryza sativa* L.) exhibiting superior quality [12,13]. These findings, consistent with those of Aoki et al. (2020), further indicate that rice protein content does not significantly affect bread volume [14]. Additionally, doughs prepared with high-amylose rice flours exhibited greater fermentation stability compared with low-amylose counterparts. Collectively, these results highlight the critical role of amylose in stabilizing dough and enhancing loaf volume, underscoring its importance in the development of gluten-free rice bread.

Beyond amylose, recent efforts to improve gluten-free bread have focused on substituting or mimicking the gluten network. Among these approaches, hydrocolloids are the most widely used and well-documented additives [15,16,17]. In gluten-free systems, hydrocolloids form gel-like networks that enhance dough cohesiveness and viscoelasticity, stabilize gas cells, bind water, and delay starch retrogradation, thereby improving fermentation expansion and overall bread quality [17]. Commonly applied hydrocolloids include hydroxypropyl methylcellulose (HPMC), carboxymethyl cellulose, β-glucan, pectin, carrageenan, xanthan gum, guar gum, locust bean gum, tara gum, and agarose [18,19], with HPMC being the most frequently employed in gluten-free bread formulations [20]. Furthermore, Foschia et al. (2016) reported the widespread incorporation of dietary fibers, with psyllium being the most common (74.1%), followed by rice bran extract (18.5%) and millet flakes (11.1%) [20]. Adequate water is essential when fibers are added to ensure appropriate crumb structure and complete starch gelatinization. Due to their gelling, water-binding, and structure-building properties, fibers increase dough viscosity, improve gas retention, and ultimately enhance loaf volume [15]. These findings suggest that, aside from psyllium, most hydrocolloids used as wheat protein substitutes in gluten-free bread remain conventional food additives.

In line with the growing global clean-label trend [21], consumer demand for natural and transparent food-grade hydrocolloids is increasing. Konjac glucomannan (K), a fiber-rich hydrocolloid extracted from konjac, has attracted considerable attention due to its multiple health benefits, including lowering cholesterol, triglycerides, and blood glucose levels, as well as supporting gut and immune health [22,23]. Owing to its excellent water-holding capacity and stability, konjac glucomannan has been widely applied as a dietary supplement in the management of obesity, diabetes, and skin disorders [24], and has also been identified as a promising candidate for gluten-free baked goods [25,26].

In our previous study, gluten-free rice bread prepared from TS17 flour with 92% water exhibited desirable appearance but poor storage stability, primarily due to rapid staling, likely associated with its high amylose content and low moisture [13]. Moreover, TS17 flour is deficient in dietary fiber and bioactive compounds. To overcome these limitations, the present study investigated the partial substitution of TS17 flour with indica black rice (B) flour, which is rich in dietary fiber and bioactive compounds [27], in combination with varying water levels and food-grade konjac powder (K), to assess their effects on rice bread quality. The physicochemical properties of TS17 and B flours were first characterized, including color parameters, particle size distribution, damaged starch, apparent amylose content, proximate composition, total dietary fiber, and water-holding capacity. Flour extracts were further analyzed for yield, bioactive constituents (total phenolics and total anthocyanins), and antioxidant activities (DPPH radical scavenging, reducing power, and ferrous ion chelation). Based on preliminary experiments, 15–25% of TS17 flour was replaced with B flour under water levels of 92%, 107%, and 122%, and bread quality was evaluated in terms of batter volume, specific gravity, and loaf characteristics (appearance, color, weight, volume, specific volume, hardness, and staling rate). The effects of K supplementation at 0–3% were also examined.

## 2. Materials and Methods

### 2.1. Materials and Reagents

**Figure 1:**
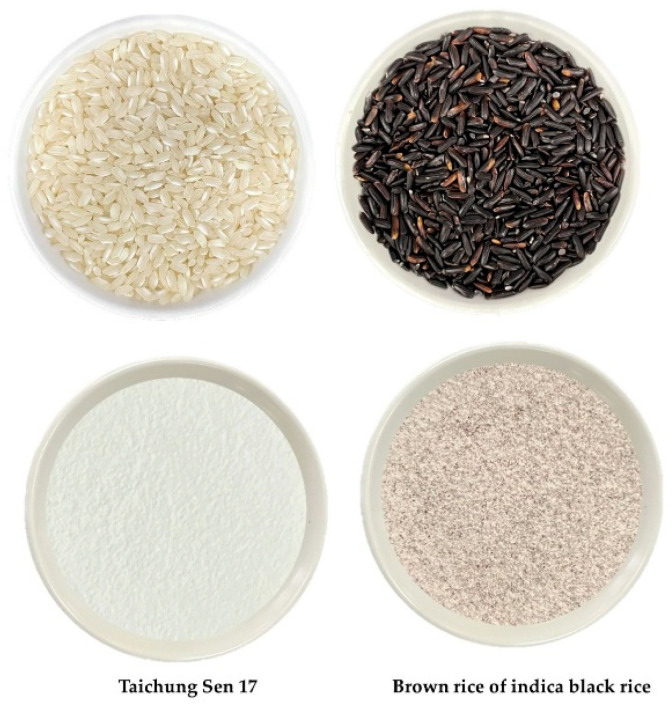
The appearance and color of rice and its flour.

Two rice varieties, Taichung Sen 17 (TS17) and indica black rice (B), were cultivated in Changhua, Taiwan. Rice flour was prepared by Hwei-Hsin Husk Rice Factory (Yunlin, Taiwan). Briefly, the rice grains were washed and soaked in a rice washing machine (DH903-605, Ding-Han Machinery Co., Ltd., New Taipei, Taiwan) with water circulation at a rice-to-water ratio of 1:6 (*w*/*w*). Soaking was conducted at room temperature (20–25 °C) until the moisture content of the grains reached 60–80%. The soaked rice was drained and milled using a jet mill (FPM-150S, Unireal Technology Co., Ltd., Taoyuan, Taiwan), followed by hot air drying at 60–70 °C. The dried flour was then sieved through a 100-mesh screen to obtain fine rice flour (Figure 1). Other ingredients, including   Saf-Instant® Gold yeast (Lesaffer, Marcq-en-Baroeul, France), unsalted butter (Fonterra Brands, New Young, Taipei, Taiwan), sucrose (Taiwan Sugar Co., Ltd., Tainan, Taiwan), sodium chloride (Taiyen Biotech Co., Ltd., Tainan, Taiwan), konjac gum (Hubei Yizhi Konjac Biotechnology Co., Ltd., Yichang, China), were obtained from commercial suppliers.

Gallic acid, Folin–Ciocalteu phenol reagent, 1,1-diphenyl-2-picrylhydrazyl (DPPH), potassium ferricyanide, ferric chloride hexahydrate, ferrozine, trichloroacetic acid, amylose (type III from potato), amylopectin (from potato), heat-stable α-amylase, protease (from *Bacillus licheniformis*), amyloglucosidase, and Celite filter aid were obtained from Sigma-Aldrich (Saint Louis, MO, USA). Ferrous chloride tetrahydrate was purchased from Alfa Aesar (Ward Hill, MA, USA). A starch damage assay kit was obtained from Megazyme International Ireland Ltd. (Wicklow, Ireland). Hexane and ethanol were purchased from Echo Chemical Co., Ltd. (Miaoli, Taiwan), while methanol, acetone, and acetonitrile were obtained from Avantor Performance Materials (Center Valley, PA, USA). Cupric sulfate, anhydrous sodium carbonate, and sodium hydroxide were obtained from Shimakyu’s Pure Chemicals (Osaka, Japan). Methylene blue was purchased from Katayama Chemical Industries (Osaka, Japan), and methyl red (pure) was obtained from Koch Light Research Laboratories (Gauteng, South Africa). Potassium iodide was purchased from Wako Pure Chemical Industries (Osaka, Japan), and potassium sulfite and iodine were purchased from Nihon Shiyaku Reagent Co. (Kanto, Japan). Hydrochloric acid, sulfuric acid, sodium dihydrogen phosphate (monobasic), and disodium phosphate dodecahydrate were obtained from Union Chemical (Hsinchu, Taiwan).

### 2.2. Experimental Design and Rice Bread Preparation

**Figure 2:**
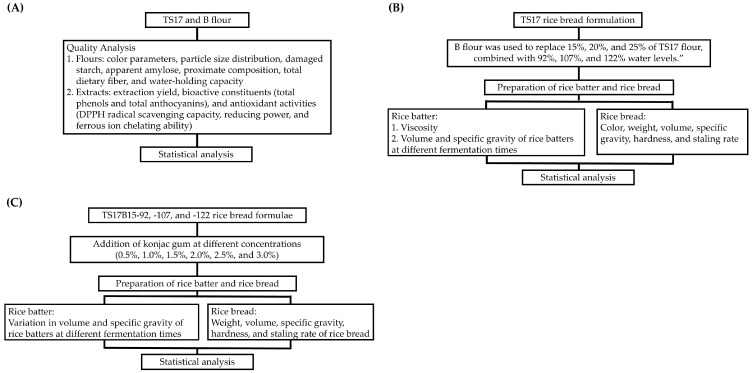
Schematic representation of the experimental design illustrating the effects of indica black rice (B), konjac powder (K), and water levels on the quality attributes of gluten-free Taichung Sen 17 (TS17) rice bread. (**A**) Assessment of the physicochemical properties of rice flours and the bioactive components and antioxidant activities of their extracts. (**B**) Effects of partial substitution of TS17 flour with B flour, combined with varying water levels, on the quality characteristics of rice batter and bread. (**C**) Effects of different concentrations of konjac powder and water levels on rice batter and bread quality.

The experimental design of this study is summarized in Figure 2. First, the physicochemical properties of TS17 and B flours—including color parameters (L*, a*, b*, WI*), particle size distribution, damaged starch, apparent amylose, proximate composition, total dietary fiber, and water-holding capacity—were characterized. In addition, their extracts were analyzed for bioactive compounds (total phenols and total anthocyanins) and antioxidant activities (DPPH radical scavenging capacity, reducing power, and ferrous ion chelating ability) to assess compositional and functional differences (Figure 2A). Second, the TS17-92 bread formulation from a previous study [13] was used as the control, with baking percentages of TS17 flour, sugar, sodium chloride, yeast, unsalted butter, and water set at 100, 10, 1.5, 1, 6, and 92%, respectively (Figure 2B). TS17 flour was partially replaced with B flour at 15%, 20%, and 25%, in combination with varying water levels (92%, 107%, and 122%) to evaluate their effects on rice bread quality. The corresponding formulations are presented in Table 1. The resulting breads were designated as TS17B15-92, TS17B20-92, TS17B25-92, TS17B15-107, TS17B20-107, TS17B25-107, TS17B15-122, TS17B20-122, and TS17B25-122. Third, the effects of konjac powder (K) at concentrations of 0.5–3.0% were examined on the quality of rice breads prepared with varying water levels (TS17B15-92, TS17B15-107, and TS17B15-122) (Figure 2C). The corresponding formulations are presented in Table 2.

Rice bread was prepared as follows: water, sugar, and salt were added to a mixing bowl and blended until homogeneous; rice flour and yeast were subsequently incorporated and mixed. When B flour and/or konjac gum (K) were included, they were added together with the rice flour during this step. The mixture was blended using a mixer (HL-11007, San Neng Bake Ware Co., Ltd., Taichung, Taiwan) at speed 1 (193 rpm) for 2 min, followed by speed 2 (253 rpm) for 4 min. Unsalted butter was then added and mixed at speed 1 for 2 min and speed 2 for 2 min, yielding a rice batter at 28–30 °C. A 650 g portion of batter was transferred into loaf pans (115 mm × 115 mm × 115 mm, SN2050, San Neng Bake Ware Co., Ltd., Taichung, Taiwan) and fermented in an incubator (PAN-RA16, Chung Pu Baking Machinery Co., Ltd., Taichung, Taiwan) at 38 °C and 80% relative humidity for 50 min. The pans were then baked in a preheated electric oven (K35E, Chung Pu Baking Machinery Co., Ltd., Taichung, Taiwan) at 230 °C (upper and lower heat) for 35 min. After baking, the loaves were removed from the pans, cooled to room temperature (24–27 °C), weighed, and subsequently packed in polyethylene bags for quality analyses. Ten batches (two loaves per batch) were prepared.

### 2.3. Determination of Physicochemical Characteristics of Rice Flours, and Bioactive Components and Antioxidant Property in Their Extracts

The reflective color of rice flours was measured using a spectrophotometer (CM-5, Konica Minolta Sensing, Inc., Tokyo, Japan) under a D65 illuminant at a 10° viewing angle. Color parameters (CIE L*, a*, b*) were recorded, and the whiteness index (WI*) was calculated as 100 − [(100 − L*)^2^ + a*^2^ + b*^2^]^1/2^, respectively. Particle size distribution was determined using a laser scattering analyzer (LA-960, Horiba Ltd., Kyoto, Japan) in dry mode. Damaged starch, apparent amylose, moisture, crude fat, crude protein, and ash were analyzed following the methods of the American Association of Cereal Chemists [28], with a nitrogen conversion factor of 5.95 for protein calculation. Carbohydrate content (g/100 g) was 100% minus those of moisture, fat, protein, and ash. Total dietary fiber (TDF) was determined according to AOAC method 985.29 [29], and water-holding capacity (g H_2_O/g sample) was measured following a previously reported method [30]. All analyses were carried out in triplicate, except for color analysis, which was performed in six replicates.

For extraction, 15 g of each rice flour was mixed with 150 mL of 50% (*v*/*v*) aqueous ethanol and incubated in a shaking water bath (75 °C, 150 rpm; SB302, Kansin Instruments, Kaohsiung, Taiwan) for 30 min. The mixture was centrifuged (Himac CR21G, Hitachi High-Technologies Co. Ltd., Tokyo, Japan; 8000 rpm, 10 min; 8060× *g*) and filtered through Advantec No. 1 filter paper (Toyo Roshi Kaisha, Ltd., Tokyo, Japan). The residue was extracted a second time with 150 mL of 50% aqueous ethanol under the same conditions. Combined extracts were rotary-evaporated at 45 °C, freeze-dried, and stored at −25 °C until further analysis. All extractions were conducted in triplicate.

Total phenols content was determined using the Folin–Ciocalteu method [31]. Sample solutions were prepared by dissolving 0.8 g of TS17 flour extract or 0.1 g of B flour extract in methanol to a final volume of 10 mL, followed by ultrasonic treatment (53 kHz, 15 min) and centrifugation (3500 rpm, 5 min; 1998× *g*). A 0.1 mL aliquot of the supernatant was mixed with 2.5 mL deionized water and 0.1 mL of Folin–Ciocalteu reagent (2.1 N), allowed to react for 6 min, and then 0.5 mL of 20% Na_2_CO_3_ solution was added. The mixture was brought to 10 mL with deionized water, incubated for 30 min, and absorbance was measured at 760 nm (U-5100, Hitachi High-Technologies Co. Ltd., Tokyo, Japan). Total phenols content was expressed as mg gallic acid equivalents (GAE)/g lyophilized extract based on a gallic acid standard curve (760 nm absorbance = 0.0009 C_gallic acid_ (µg/mL) + 0.0069, R^2^ = 0.9992). Analyses were performed in triplicate.

Total anthocyanins content was determined using the pH-differential method [27]. Briefly, 200 mg of extract was mixed with 7 mL methanol–1 M HCl (85:15, *v*/*v*), subjected to ultrasonic treatment (53 kHz, 5 min) in the dark, and diluted to 10 mL with methanol–1 M HCl. Results were expressed as µg cyanidin-3-glucoside equivalents (C3GE) per gram of lyophilized extract. Measurements were performed in triplicate.

Antioxidant activities were evaluated as follows. DPPH radical scavenging ability was determined for extract concentrations of 0–0.5 mg/mL [32]. Reducing power was measured for methanolic extract solutions at 0–1.25 mg/mL (rice flour) and 0–25 mg/mL (bread) [32]. Ferrous ion chelating activity was assessed for extract concentrations of 0–5 mg/mL (rice flour) [32]. The effective concentration for 50% DPPH scavenging (EC_50_), reducing power at absorbance 0.5, and 50% ferrous ion chelation were calculated. EC_50_ values for TS17 flour extract DPPH scavenging were obtained by extrapolation of linear regression; other EC_50_ values were determined by interpolation of linear regression. All assays were conducted in triplicate.

### 2.4. Physicochemical Property of Rice Batter

Rice batter (400 mL) was transferred into a 500 mL beaker, and its viscosity was measured at 10 rpm using an LV-4 spindle on a rotational viscometer (DV2TLV, Ametek Brookfield Co. Ltd., Middleborough, MA, USA). To evaluate fermentation expansion, 650 g of rice batter was placed in a 3 L graduated cylinder and incubated at 38 °C with 80% relative humidity. Batter volume was recorded at 10 min intervals throughout the fermentation period. Specific gravity at each time point was calculated by dividing the weight of 650 g of rice batter by the weight of an equal volume of water. All measurements were performed in triplicate.

### 2.5. Physical Characteristics of Rice Bread

Bread weight was measured using a precision balance (GG4002-S, Mettler-Toledo, Langacher Greifense, Switzerland). Moisture content was determined according to the method of the American Association of Cereal Chemists [28]. Bread volume was measured using the rapeseed displacement method [33], and specific volume was calculated by dividing the bread volume by its weight. The color of the bread crust and crumb was evaluated using a spectrophotometer (CIE L*, a*, b*). Hue angle (h*) and chroma (c*) were calculated as h* = arctan(b*/a*) and c* = (a*^2^ + b*^2^)^1/2^, respectively. The total color difference (Δ*E*) between the control and sample was calculated as *∆E* = [(L*_c_ − L*_s_)^2^ + (a*_c_ − a*_s_) ^2^ + (a*_c_ − a*_s_)^2^]^1/2^, where L*, a*, and b* represent the color coordinates of the control (c) and sample (s). Bread hardness was measured at the center of a 2.5 × 2.5 × 2.5 cm sample using a texture analyzer (TA-XT2, 25 kg model; Stable Micro Systems, Surrey, UK) with a P45 probe. The sample was compressed to 50% of its original height at 1.0 mm/s. Test parameters were as follows: pre-test speed 1 mm/s; test speed 5 mm/s; post-test speed 5 mm/s; compression distance 10 mm; test duration 5 s; trigger force 5 g. Hardness was measured immediately after baking and after 24 h of storage at room temperature. The staling rate was calculated as [34]

$$\mathrm{Staling}\;\mathrm{rate}=\left(\frac{\mathrm{crumb}\;\mathrm{hardness}\;\mathrm{after}\;24\;h\;\left[N\right]-\mathrm{crumb}\;\mathrm{hardness}\;\mathrm{after}\;2\;h\;\left[N\right]}{\mathrm{hardness}\;\mathrm{after}\;2\;h\;\left[N\right]}\right)$$

Color and hardness measurements were performed in six replicates, while other quality parameters were analyzed in triplicate.

### 2.6. Statistical Analysis

All measurements were performed in triplicate (*n* = 3), except for color and texture analyses, which were conducted in six replicates (*n* = 6). Experimental data were subjected to analysis of variance (ANOVA) using the SAS 9.4 statistical software package (SAS Institute, Cary, NC, USA). Significant differences among means were determined using Duncan’s multiple range test at a significance level of 0.05.

## 3. Results and Discussion

### 3.1. Physicochemical Characteristics of Rice Flours, and Bioactive Components and Antioxidant Property in Their Extracts

**Figure 3:**
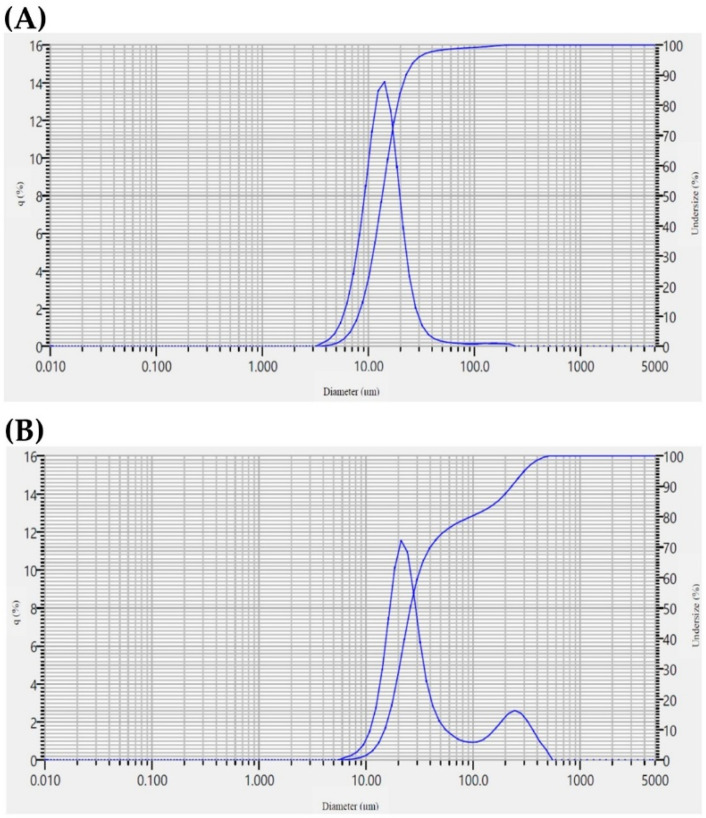
Particle size distribution of the rice flour. (**A**) TS17 flour. (**B**) B flour.

The physicochemical properties of TS17 and B rice flours exhibited notable differences. TS17 flour demonstrated significantly higher L* and whiteness index (WI*) values, indicating greater brightness and overall whiteness, whereas B flour showed elevated a* and b* values, reflecting stronger red and yellow color intensities (Table 3). Particle size analysis revealed median sizes of 13.51 μm for TS17 flour and 25.93 μm for B flour, with the larger particle size of B likely attributed to the presence of bran layers (Figure 3, Table 3). Correspondingly, the damaged starch content was markedly higher in TS17 flour (5.30%) than in B flour (0.19%), consistent with previous reports indicating that smaller particle size under identical milling conditions leads to higher damaged starch content [35,36,37]. Low damaged starch content, intact starch granules, and moderate particle size are considered advantageous for gluten-free rice bread production [37]. TS17 flour also exhibited significantly higher apparent amylose content (30.47%) than B flour (9.89%), classifying TS17 as a high-amylose variety and B as low-amylose. In contrast, B flour showed superior water-holding capacity, likely due to its higher total dietary fiber content. In addition, B flour contained significantly higher levels of moisture, protein, fat, ash, and total dietary fiber compared to TS17 (Table 3), attributable to bran retention [38,39,40].

**Figure 4:**
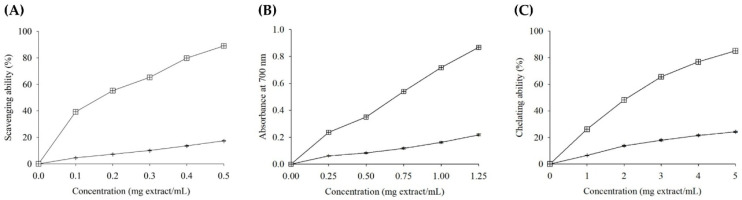
Antioxidant property of lyophilized rice flour extracts. (**A**) Scavenging ability on DPPH radicals. (**B**) Reducing power. (**C**) Chelating ability on ferrous ions. Each value is expressed as mean ± standard deviation (*n* = 3). TS17 (✢), B (田).

Extraction yield is a widely applied indicator for assessing the efficiency of solvents in isolating target compounds from raw materials. The extraction yield obtained from B was significantly greater than that from TS17 (Table 3). Total phenolic compounds, which are well recognized for their antioxidant, anti-inflammatory, antibacterial, and cardioprotective properties [41], were present at significantly higher levels in the B extract compared to the TS17 extract (Table 3). Notably, anthocyanins—bioactive compounds associated with diverse health-promoting effects, including antioxidant, anti-inflammatory, antidiabetic, and potential anticancer activities [42,43,44]—were exclusively detected in the B extract, with no presence observed in the TS17 extract. The predominant anthocyanins identified in black rice are cyanidin-3-glucoside and peonidin-3-glucoside [45]. With the increasing concentrations of B and TS17 extracts, their DPPH radical scavenging activity, reducing power, and ferrous ion chelating ability were significantly enhanced (Figure 4). At the same concentration, the B extract exhibited markedly stronger antioxidant activity than the TS17 extract, which could be attributed to its higher levels of bioactive compounds (Table 3). A further comparison of EC_50_ values (mg extract/mL) showed that the B extract yielded values of 0.17, 0.70, and 2.10 for DPPH radical scavenging, reducing power, and ferrous ion chelation, respectively, which were substantially lower than those of the TS17 extract (3.12, 3.14, and 10.69). Collectively, these findings clearly demonstrate that the B extract possesses significantly superior antioxidant potential compared with TS17.

### 3.2. Quality Characteristics of the Rice Batter and Rice Bread Made by Replacing Part of TS17 with B

#### 3.2.1. Quality Characteristics of the Rice Batter

**Figure 5:**
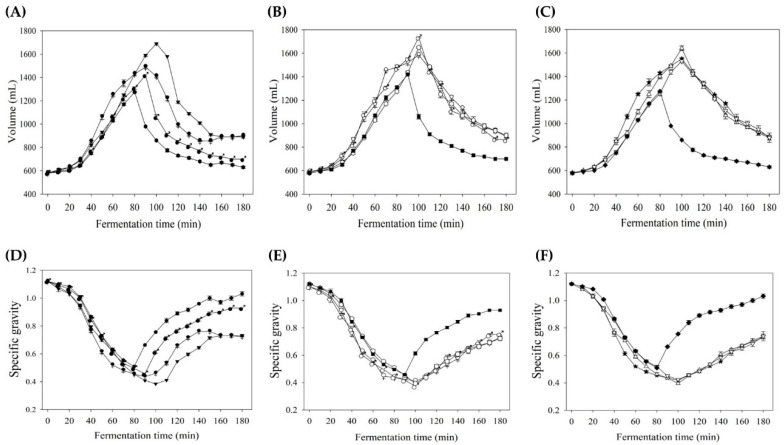
Variation in volume and specific gravity of rice batters made by partially replacing TS17 flour with B flour at different fermentation times. (**A**–**C**) Volume measurements of rice batters with 92%, 107%, and 122% water addition, respectively; (**D**–**F**) specific gravity measurements of the same batter formulations. Each value is expressed as mean ± standard deviation (*n* = 3). TS17-92 (▼), TS17B15-92 (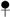), TS17B20-92 (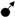), TS17B25-92 (⬣). TS17-107 (■), TS17B15-107 (♀), TS17B20-107 (♂), TS17B25-107 (⬡). TS17-122 (◆), TS17B15-122 (★), TS17B20-122 (△), TS17B25-122 (▽).

With increasing levels of B flour substitution in TS17, the viscosity of all samples was significantly enhanced, with TS17B25-92 exhibiting the highest viscosity at 16,787 cp (Table 4). This increase in viscosity is likely associated with the starch type and content, total dietary fiber, and water-holding capacity of B flour (Table 3). The maximum volumes of rice batter under varying water addition levels and B flour substitution ratios are presented in Figure 5. At 92% water addition (Figure 5A), TS17-92 displayed the highest batter volume of 1690 mL; however, as the B flour substitution level increased, batter volume gradually declined, suggesting that excessive replacement with B flour may hinder batter expansion. At 107% water addition (Figure 5B), TS17B20-107 achieved the highest volume of 1735 mL, while at 122% water addition (Figure 5C), TS17B20-122 reached the maximum volume of 1640 mL. Figure 5D–F illustrate the specific gravity of the rice batters, revealing a clear inverse relationship between batter volume and specific gravity, whereby higher volumes corresponded to lower specific gravity. Regarding starch composition, amylose contributes to moldability, texture modulation, and gel formation, whereas amylopectin enhances anti-staling properties, freezing stability, thickening, and water retention, thus offering considerable utility in food processing and related industries [46]. Collectively, these findings indicate the existence of an optimal B flour substitution level for maximizing batter expansion, which is likely closely related to amylose content and may vary depending on the water content.

#### 3.2.2. Quality Characteristics of the Rice Bread

**Figure 6:**
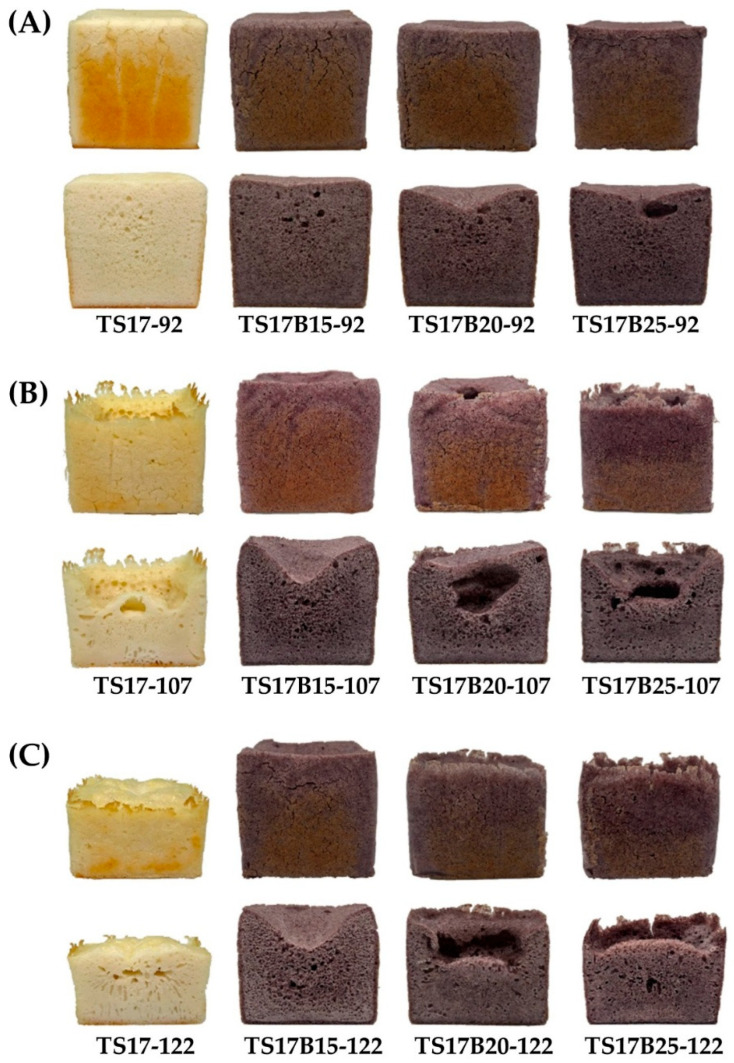
The appearance and color of rice breads made by replacing part of TS17 flour with B flour. (**A**) 0–25% B flour replacement levels with 92% water addition. (**B**) 0–25% B flour replacement levels with 107% water addition. (**C**) 0–25% B flour replacement levels with 122% water addition.

Figure 6 shows the appearance and color of rice breads in which B flour partially replaced TS17 flour. Under the same water addition level, increasing the proportion of B flour substitution resulted in a darker crumb color (lower L* value), reduced yellowness (lower b* value), and an increase in red-purple intensity (higher h* value), as summarized in Table 5. When the substitution level of B flour was held constant, increasing the amount of water added led to decreases in redness (a* value) and chroma (c* value) of the bread crumb. This may be due to the dilution effect associated with higher moisture levels in the formulation. All rice breads containing B flour, as well as the control, had Δ*E* values greater than 3, indicating that the color differences were perceptible to the human eye [47].

At the same level of water addition, the weight of rice bread samples increased with higher substitution levels of B flour (Table 6). This can be attributed to the higher total dietary fiber content and water holding capacity of B flour compared to TS17 flour (Table 3), which made the moisture in the batter more resistant to evaporation during baking. Conversely, when the proportion of B flour replacement was held constant, the weight of rice breads decreased as water addition increased. This is because 650 g of batter was placed into each mold, and batters with higher water content lost more moisture during baking.

Regarding volume, under 92% water addition, TS17B15-92 and TS17-92 exhibited the highest bread volumes, reaching 1042 mL and 1032 mL, respectively (Table 6). At 107% water addition, the highest volume was observed in TS17B15-107 (917 mL). Under 122% water addition, the highest volumes were found in TS17B15-122 (775 mL) and TS17B20-122 (761 mL). As the replacement level of B flour increased, the appearance of the rice bread became more concave (Figure 6). Although the fermented batter reached the mold height of 10.5 cm before baking, the low amylose concentration reduced its gelling capacity. This compromised the batter’s ability to support its structure during baking, leading to collapse. The specific volume of rice bread, a critical quality indicator [48], showed a positive correlation with total bread volume in this study (Table 6).

After baking, the hardness of the bread samples decreased as the proportion of B flour increased (Table 6). Among all bread samples stored at room temperature for 24 h, TS17B25-122 exhibited the lowest hardness, although it was still significantly higher than its counterpart measured after 2 h of cooling. Additionally, TS17B25-107 showed the slowest staling rate (1.04) (Table 6). These results indicate that partially replacing TS17 flour with B flour and adjusting water content appropriately can effectively delay the retrogradation of gluten-free rice bread.

To mitigate the adverse effects of starch retrogradation on texture during storage, this study sought potential solutions. According to several studies, hydrocolloids such as HPMC, carboxymethyl cellulose, β-glucan, pectin, carrageenan, xanthan gum, guar gum, locust bean gum, tara gum, and agarose can improve the texture of gluten-free bread [18,19]. These ingredients enhance the viscoelastic properties and water retention of baked goods, thereby improving texture and extending shelf life [49]. In addition, hydrocolloids can increase rice batter viscosity and improve gas retention during fermentation, resulting in greater bread volume [50]. However, many of these hydrocolloids are considered food additives. To align with the clean label concept, this study used K powder as a natural, food-grade hydrocolloid with documented health benefits [51]. TS17B15-92, TS17B15-107, and TS17B15-122 were selected as test samples to evaluate the effects of adding 0.5%, 1.0%, 1.5%, 2.0%, 2.5%, and 3.0% konjac powder on the quality characteristics of rice bread.

### 3.3. Effect of Konjac Gum on the Quality of Rice Batter and Rice Bread

#### 3.3.1. Quality Characteristics of the Rice Batter

After mixing the raw materials, all rice batters containing K exhibited viscosities beyond the highest measurable limit of the viscometer used in this study (60,000 cp). This substantial increase in viscosity can be attributed to the total dietary fiber content of K, which enhances the water-holding capacity, viscosity, and structural properties of the rice batter, as previously reported [50]. The water-holding capacity of K was determined to be 95.52 g H_2_O absorbed/g sample, which is considerably higher than that of TS17 and B flours (Table 3).

**Figure 7:**
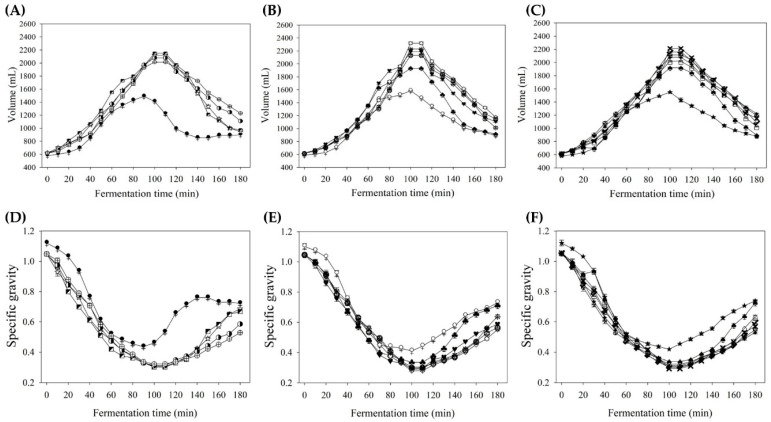
Variation in volume (**A**–**C**) and specific gravity (**D**–**F**) of rice batters with various concentrations of konjac gum added at different fermentation times. (**A**,**D**) 92% water addition. (**B**,**E**) 107% water addition. (**C**,**F**) 122% water addition. Each value is expressed as mean ± standard deviation (*n* = 3). TS17B15-92 (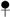), TS17B15K0.5-92 (✩), TS17B15K1.0-92 (◩), TS17B15K1.5-92 (◑), TS17B15K2.0-92 (⊕), TS17B15-107 (♀), TS17B15K0.5-107 (♣), TS17B15K1.0-107 (♥), TS17B15K1.5-107 (◇), TS17B15K2.0-107 (❋), TS17B15K2.5-107 (❂), TS17B15-122 (★), TS17B15K0.5-122 (♠), TS17B15K1.0-122 (⌂), TS17B15K1.5-122 (✔), TS17B15K2.0-122 (✖), TS17B15K2.5-122 (✠), TS17B15K3.0-122 (✦).

As a result, the bread volumes in the experimental groups with 92%, 107%, and 122% water additions were all significantly greater than their respective controls (Figure 7A–C). This aligns with previous findings that the incorporation of hydrocolloids during mixing enhances batter viscosity and gas retention, contributing to greater bread volume [50]. During fermentation, regardless of the water addition level, K improved gas retention and helped maintain batter structure. The highest volumes in the experimental groups were observed at 100 to 110 min of fermentation, and the structural stability was notably better than that of the controls (TS17B15-92, TS17B15-107, and TS17B15-122). In addition, changes in the specific gravity of rice batter (Figure 7D,E) across different K concentrations and fermentation times revealed an inverse relationship between specific gravity and batter volume.

#### 3.3.2. Quality Characteristics of Rice Bread

**Figure 8:**
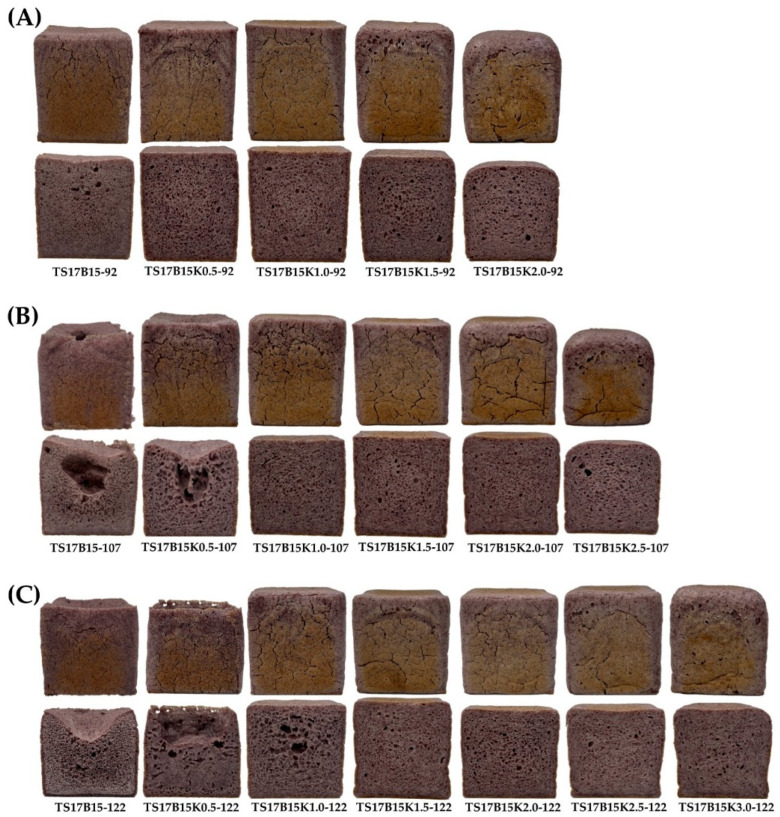
The appearance and color of TS17B15 rice bread with different concentrations of konjac gum and varying water levels. (**A**) 0–2% K with 92% water. (**B**) 0–2.5% K with 107% water. (**C**) 0–3.0% K with 122% water.

Figure 8 illustrates the appearance and color of rice bread containing varying concentrations of konjac powder (K). At a constant water addition level, the sample weight increased with higher K concentration (Table 7), which is likely attributable to the higher total dietary fiber content and water-holding capacity of K compared with TS17 and B flours (Table 3), thereby reducing water loss during baking. The highest bread volumes achieved under each water addition level were 1062 mL for TS17B15K1.0-92, and 1063 mL for both TS17B15K1.5-107 and TS17B15K2.0-122. These results suggest that K enhances batter viscosity, fermentation, and gas retention, thereby improving bread volume [50,52]. Espert et al. (2017) reported that certain hydrocolloids, such as HPMC, carboxymethyl cellulose, and methylcellulose, possess both hydrophilic and hydrophobic groups [53]. These properties improve interfacial activity and form gel networks during baking, reinforcing dough structure, enhancing gas retention, and increasing bread volume.

The hardness of baked bread prepared with K-added rice batters was significantly lower than that of the control after 2 h of cooling at room temperature (Table 7). However, bread hardness increased with increasing K concentration. After 24 h at room temperature, the hardness of all breads increased, yet those with K remained significantly softer than the control. This is consistent with findings by Nakamura et al. (2016), who reported that adding konjac powder (0.25–0.75%) significantly reduced the hardness of rice bread, indicating that konjac softens bread compared to rice flour alone [26]. Regarding bread staling, the slowest staling rates under each water addition level were observed in TS17B15K1.0-92 (1.10), TS17B15K1.5-107 (1.29), and TS17B15K2.0-122 (1.15) (Table 7). However, excessive K may accelerate staling due to insufficient moisture. The use of hydrocolloids is critical in gluten-free breadmaking. They stabilize bread structure, retain moisture, and delay starch retrogradation, improving product stability during storage or freezing. Due to their high water-binding capacity, formulas with hydrocolloids require more water [54,55]. Additionally, hydrocolloids enhance the viscoelasticity, texture, and shelf life of baked products [49]. Nonetheless, variations in hydrocolloid type and dosage can influence dough rheology and bread texture, ultimately affecting the final quality of gluten-free baked products [54]. Therefore, the proportion of hydrocolloid appears to play a critical role in shaping sensory characteristics. Finally, TS17B15K1.0-92, TS17B15K1.5-107, and TS17B15K2.0-122 were identified as the recommended formulations in this study, as they exhibited the highest bread volumes, lower staling rates, and desirable crumb structures.

## 4. Conclusions

This study demonstrates that partially replacing Taichung Sen 17 rice flour with indica black rice flour, together with appropriate water adjustment, can effectively enhance the quality of gluten-free rice bread by increasing loaf volume and delaying starch retrogradation. This substitution significantly enhances antioxidant capacity and enriches the nutritional profile, particularly in phenolic compounds. The natural hydrocolloid konjac gum further improves dough structure, moisture retention, and overall bread quality. However, the excessive addition of konjac gum may adversely affect staling due to compromised moisture balance. These findings provide a practical strategy for developing functional gluten-free bakery products with improved physicochemical, nutritional, and storage characteristics. Future research should further explore the interactions between konjac gum and other hydrocolloids to optimize formulation and extend shelf life, thereby supporting innovation in health-oriented gluten-free food development.

## Figures and Tables

**Table 1:** Formulations of rice breads with partial replacement of TS17 flour by B flour.

Ingredient (%)	TS17-92 ^1^	TS17B15-92 ^2^	TS17B20-92	TS17B25-92
TS17 flour	100	85	80	75
B flour	-	15	20	25
Sugar	10	10	10	10
Sodium chloride	1.5	1.5	1.5	1.5
Yeast	1.0	1.0	1.0	1.0
Unsalted butter	6.0	6.0	6.0	6.0
Water	92	92	92	92
Total	210.2	210.5	210.5	210.5
Ingredient (%)	TS17-107	TS17B15-107	TS17B20-107	TS17B25-107
TS17 flour	100	85	80	75
B flour	-	15	20	25
Sugar	10	10	10	10
Sodium chloride	1.5	1.5	1.5	1.5
Yeast	1.0	1.0	1.0	1.0
Unsalted butter	6.0	6.0	6.0	6.0
Water	107	107	107	107
Total	225.5	225.5	225.5	225.5
Ingredient (%)	TS17-122	TS17B15-122	TS17B20-122	TS17B25-122
TS17 flour	100	85	80	75
B flour	-	15	20	25
Sugar	10	10	10	10
Sodium chloride	1.5	1.5	1.5	1.5
Yeast	1.0	1.0	1.0	1.0
Unsalted butter	6.0	6.0	6.0	6.0
Water	122	122	122	122
Total	240.5	240.5	240.5	240.5

^1^ TS17-92, TS17-107, and TS17-122 represent the control rice bread groups formulated with 92%, 107%, and 122% water (based on flour weight), respectively, using only TS17 flour. ^2^ TS17B15, TS17B20 and TS17B25: rice breads made by replacing 15%, 20% and 25% of TS17 flour with B flour, respectively. 92, 107 and 122 represent the baking percentages with added water.

**Table 2:** Rice bread formulations with B flour substitution and konjac gum addition.

Ingredient (%)	TS17B15K0.5-92 ^1^	TS17B15K1.0-92	TS17B15K1.5-92	TS17B15K2.0-92		
TS17 flour	85	85	85	85		
B flour	15	15	15	15		
Sugar	10	10	10	10		
Sodium chloride	1.5	1.5	1.5	1.5		
Yeast	1.0	1.0	1.0	1.0		
Unsalted butter	6.0	6.0	6.0	6.0		
Water	92	92	92	92		
Konjac gum (K)	0.5	1.0	1.5	2.0		
Total	211.0	211.5	212.0	212.5		
Ingredient (%)	TS17B15K0.5-107	TS17B15K1.0-107	TS17B15K1.5-107	TS17B15K2.0-107	TS17B15K2.5-107	
TS17 flour	85	85	85	85	85	
B flour	15	15	15	15	15	
Sugar	10	10	10	10	10	
Sodium chloride	1.5	1.5	1.5	1.5	1.5	
Yeast	1.0	1.0	1.0	1.0	1.0	
Unsalted butter	6.0	6.0	6.0	6.0	6.0	
Water	107	107	107	107	107	
Konjac gum (K)	0.5	1.0	1.5	2.0	2.5	
Total	226.0	226.5	227.0	227.5	228.0	
Ingredient (%)	TS17B15K0.5-122	TS17B15K1.0-122	TS17B15K1.5-122	TS17B15K2.0-122	TS17B15K2.5-122	TS17B15K3.0-122
TS17 flour	85	85	85	85	85	85
B flour	15	15	15	15	15	15
Sugar	10	10	10	10	10	10
Sodium chloride	1.5	1.5	1.5	1.5	1.5	1.5
Yeast	1.0	1.0	1.0	1.0	1.0	1.0
Unsalted butter	6.0	6.0	6.0	6.0	6.0	6.0
Water	122	122	122	122	122	122
Konjac gum (K)	0.5	1.0	1.5	2.0	2.5	3.0
Total	241.0	241.5	242.0	242.5	243.0	243.5

^1^ TS17B15K0.5, TS17B15K1.0, TS17B15K1.5, TS17B15K2.0, TS17B15K2.5 and TS17B15K3.0 were formulated with 0.5%, 1.0%, 1.5%, 2.0%, 2.5% and 3.0% (*w*/*w*) konjac gum, respectively. 92, 107 and 122 represent the baking percentages with added water.

**Table 3:** Physicochemical quality characteristics of rice flour and its extract.

	TS17 ^1^	B
L*	97.46 ± 0.15 a ^3^	76.75 ± 0.04 b
a*	0.17 ± 0.01 b	2.85 ± 0.02 a
b*	2.81 ± 0.05 b	3.93 ± 0.06 a
WI*	96.21 ± 0.13 a	76.25 ± 0.04 b
Median size (μm)	13.51 ± 0.41 b	25.93 ± 0.58 a
Damaged starch (%)	5.30 ± 0.18 a	0.19 ± 0.02 b
Apparent amylose (%, dry basis)	30.47 ± 0.22 a	9.89 ± 0.17 b
Water holding capacity (g H_2_O absorbed/g sample)	0.96 ± 0.01 b	1.24 ± 0.02 a
Moisture (g/100 g sample)	5.06 ± 0.01 b	8.08 ± 0.02 a
Crude protein (g/100 g sample)	7.89 ± 0.03 b	8.73 ± 0.04 a
Crude fat (g/100 g sample)	0.35 ± 0.02 b	0.71 ± 0.05 a
Crude ash (g/100 g sample)	0.28 ± 0.03 b	1.06 ± 0.05 a
Carbohydrate (g/100 g sample)	86.42 ± 0.08 a	81.42 ± 0.11 b
Total dietary fiber (g/100 g sample)	1.51 ± 0.02 b	3.36 ± 0.02 a
**Rice flour extract**	**TS17** ^1^	**B**
Yield (g extract/100 g powder)	0.80 ± 0.02 b	3.71 ± 0.12 a
Total phenols (mg GAE ^2^/g lyophilized extract)	4.30 ± 0.16 b	42.52 ± 0.28 a
Total anthocyanin content (mg C3GE ^2^/g lyophilized extract)	nd	1.26 ± 0.01 a

^1^ TS17: Taichung Sen 17. B: Indica black rice. ^2^ GAE: gallic acid equivalent. C3GE: Cyanidin-3-glucoside equivalent. ^3^ Each value is expressed as mean ± standard deviation (*n* = 3, but *n* = 6 for color). Means with different lowercase letters within a row differ significantly (*p* < 0.05). nd: not detected.

**Table 4:** Viscosity of rice batter made by replacing part of TS17 flour with B flour.

	Viscosity (cp)
**TS17-92**	8257 ± 166 D ^1^
**TS17B15-92**	9207 ± 31 C
**TS17B20-92**	12,213 ± 122 B
**TS17B25-92**	16,787 ± 42 A
**TS17-107**	3123 ± 125 H
**TS17B15-107**	3393 ± 114 G
**TS17B20-107**	4467 ± 90 F
**TS17B25-107**	6113 ± 153 E
**TS17-122**	1417 ± 55 L
**TS17B15-122**	1427 ± 50 K
**TS17B20-122**	1953 ± 83 J
**TS17B25-122**	2620 ± 100 I

^1^ Each value is expressed as mean ± standard deviation (*n* = 3). Means with different capital letters within a column differ significantly (*p* < 0.05).

**Table 5:** Crumb color of rice bread made by replacing part of TS17 flour with B flour.

	L*	a*	b*	h* (°)	c*	Δ*E*
TS17-92	75.69 ± 1.33 B ^1^	−1.39 ± 0.05 H	8.49 ± 0.31 C		8.61 ± 0.31 C	
TS17B15-92	51.81 ± 0.82 C	6.33 ± 0.54 A	1.81 ± 0.33 FG	16.18 ± 1.48 C	6.60 ± 0.53 D	26.01 ± 1.67 E
TS17B20-92	49.47 ± 0.85 DE	5.69 ± 0.20 B	1.35 ± 0.19 HI	13.28 ± 1.54 C	5.86 ± 0.22 E	28.11 ± 1.85 D
TS17B25-92	47.24 ± 0.92 F	5.27 ± 0.24 C	1.21 ± 0.14 I	12.95 ± 1.55 C	5.41 ± 0.23 FG	30.13 ± 1.52 C
TS17-107	80.55 ± 1.70 A	−0.92 ± 0.08 G	10.91 ± 0.20 A		10.95 ± 0.20 A	
TS17B15-107	51.25 ± 0.93 C	5.26 ± 0.43 C	2.67 ± 0.12 D	27.19 ± 2.83 A	5.92 ± 0.35 E	31.10 ± 1.19 BC
TS17B20-107	49.90 ± 0.40 DE	5.14 ± 0.08 C	2.26 ± 0.09 E	23.74 ± 1.08 B	5.62 ± 0.05 EF	31.81 ± 0.68 BC
TS17B25-107	45.88 ± 1.45 G	4.60 ± 0.26 EF	1.81 ± 0.13 FG	21.41 ± 3.63 B	4.96 ± 0.28 HI	36.30 ± 2.59 A
TS17-122	80.31 ± 1.54 A	−1.14 ± 0.11 GH	9.39 ± 0.36 B		9.46 ± 0.36 B	
TS17B15-122	50.60 ± 0.54 CD	5.01 ± 0.15 CD	2.14 ± 0.44 EF	23.09 ± 4.69 B	5.48 ± 0.15 FG	31.24 ± 1.44 BC
TS17B20-122	49.10 ± 0.26 E	4.84 ± 0.12 DE	1.90 ± 0.24 FG	21.43 ± 2.70 B	5.21 ± 0.12 GH	32.68 ± 1.39 B
TS17B25-122	46.09 ± 0.42 FG	4.47 ± 0.11 F	1.65 ± 0.15 GH	20.20 ± 1.54 B	4.78 ± 0.15 I	35.57 ± 1.70 A

^1^ Each value is expressed as mean ± standard deviation (*n* = 6). Means with different capital letters within a column differ significantly (*p* < 0.05).

**Table 6:** Physical quality characteristics of rice bread made by replacing part of TS17 flour with B flour.

	Weight (g)	Volume (mL)	Specific Volume (mL/g)	Hardness (N, 2 h After Baking)	Hardness (N, 24 h After Baking)	Staling Rate
TS17-92	600.2 ± 2.05 G ^1^	1032 ± 6 A	1.72 ± 0.01 A	12.79 ± 1.63 B ^1^	30.23 ± 4.31 C ^1^	1.36 ± 0.28 E
TS17B15-92	609.3 ± 0.80 C	1042 ± 5 A	1.71 ± 0.01 A	8.09 ± 1.26 D	21.06 ± 2.63 E	1.60 ± 0.36 C
TS17B20-92	610.9 ± 0.30 B	980 ± 4 B	1.60 ± 0.01 B	7.58 ± 1.21 E	19.14 ± 2.45 F	1.53 ± 0.34 CD
TS17B25-92	611.2 ± 0.65 A	935 ± 5 C	1.53 ± 0.01 C	6.81 ± 0.67 F	18.13 ± 1.91 F	1.24 ± 0.34 F
TS17-107	594.4 ± 1.92 J	872 ± 4 E	1.47 ± 0.01 D	13.21 ± 1.09 B	52.31 ± 4.95 B	2.96 ± 0.24 A
TS17B15-107	602.2 ± 1.70 F	917 ± 3 D	1.52 ± 0.01 C	8.42 ± 0.83 CD	24.24 ± 1.42 D	1.49 ± 0.25 D
TS17B20-107	604.1 ± 1.55 E	872 ± 2 E	1.44 ± 0.01 E	7.48 ± 0.22 E	20.66 ± 1.64 E	1.18 ± 0.16 G
TS17B25-107	607.5 ± 0.70 D	812 ± 5 F	1.34 ± 0.01 F	6.88 ± 2.35 F	18.36 ± 0.57 F	1.04 ± 0.08 H
TS17-122	591.8 ± 1.81 L	642 ± 3 I	1.08 ± 0.01 I	32.28 ± 5.10 A	101.82 ± 12.49 A	2.15 ± 0.31 B
TS17B15-122	593.3 ± 1.05 K	775 ± 5 G	1.30 ± 0.01 G	8.83 ± 0.16 C	23.27 ± 1.89 D	1.64 ± 0.21 C
TS17B20-122	595.7 ± 0.50 I	761 ± 3 G	1.28 ± 0.01 G	7.59 ± 0.27 E	18.57 ± 0.95 F	1.45 ± 0.10 D
TS17B25-122	598.3 ± 1.05 H	732 ± 5 H	1.22 ± 0.01 H	6.77 ± 0.20 F	14.86 ± 0.58 G	1.19 ± 0.04 FG

^1^ Each value is expressed as mean ± standard deviation (*n* = 6 for hardness and staling rate, *n* = 3 for other qualities). Means with different capital letters within a column differ significantly (*p* < 0.05).

**Table 7:** Physical quality characteristics of rice bread with different concentrations of konjac gum added.

	Weight (g)	Moisture (%)	Volume (mL)	Hardness (N) (2 h After Baking)	Hardness (N) (24 h After Baking)	Staling Rate
TS17B15-92	609.30 ± 0.80 E ^1^	46.62 ± 0.27 O	1042 ± 5 CD	8.09 ± 1.26 C	21.06 ± 2.63 C	1.60 ± 0.36 A
TS17B15K0.5-92	612.43 ± 0.50 D	47.23 ± 0.17 N	1045 ± 5 BC	2.57 ± 0.35 H	5.63 ± 0.33 L	1.22 ± 0.25 EF
TS17B15K1.0-92	614.70 ± 0.36 C	47.83 ± 0.16 M	1062 ± 4 A	3.16 ± 0.25 G	6.60 ± 0.56 J	1.10 ± 0.07 F
TS17B15K1.5-92	616.83 ± 0.40 B	48.36 ± 0.16 L	1030 ± 6 FG	3.74 ± 0.19 F	8.90 ± 0.55 G	1.39 ± 0.17 D
TS17B15K2.0-92	618.30 ± 0.53 A	49.08 ± 0.12 K	1005 ± 5 I	4.36 ± 0.33 E	10.70 ± 0.53 F	1.46 ± 0.22 C
TS17B15-107	602.20 ± 1.70 G	48.54 ± 0.28 L	917 ± 3 K	9.72 ± 0.83 A	24.24 ± 1.42 AB	1.49 ± 0.25 BC
TS17B15K0.5-107	606.30 ± 0.72 F	48.66 ± 0.12 L	979 ± 3 J	2.17 ± 0.17 I	5.50 ± 0.31 L	1.54 ± 0.13 B
TS17B15K1.0-107	608.50 ± 1.20 E	49.11 ± 0.22 K	1045 ± 2 BC	2.71 ± 0.25 H	6.61 ± 0.56 J	1.45 ± 0.18 C
TS17B15K1.5-107	611.50 ± 1.21 D	49.98 ± 0.15 J	1063 ± 3 C	3.18 ± 0.14 G	7.27 ± 0.39 I	1.29 ± 0.07 E
TS17B15K2.0-107	614.43 ± 0.42 C	50.70 ± 0.25 I	1053 ± 5 A	3.66 ± 0.17 FG	8.95 ± 0.29 G	1.46 ± 0.16 C
TS17B15K2.5-107	617.33 ± 0.45 AB	51.26 ± 0.25 H	1005 ± 5 I	4.50 ± 0.23 DE	11.56 ± 0.65 E	1.57 ± 0.19 AB
TS17B15-122	593.25 ± 1.05 K	53.18 ± 0.13 G	775 ± 5 M	8.83 ± 0.16 B	23.27 ± 1.89 B	1.64 ± 0.21 A
TS17B15K0.5-122	595.67 ± 0.67 J	53.87 ± 0.16 F	788 ± 4 L	9.91 ± 0.53 A	25.02 ± 1.54 A	1.53 ± 0.06 B
TS17B15K1.0-122	597.90 ± 0.46 I	54.29 ± 0.16 E	1028 ± 3 G	2.54 ± 0.21 H	6.04 ± 0.60 K	1.38 ± 0.17 D
TS17B15K1.5-122	600.43 ± 0.93 H	54.93 ± 0.23 D	1035 ± 5 EF	3.22 ± 0.21 G	7.35 ± 0.61 I	1.28 ± 0.13 E
TS17B15K2.0-122	603.40 ± 0.70 G	55.33 ± 0.10 C	1063 ± 2 A	3.88 ± 0.14 F	8.35 ± 0.34 H	1.15 ± 0.07 F
TS17B15K2.5-122	606.60 ± 1.37 F	56.04 ± 0.15 B	1038 ± 2 DE	4.34 ± 0.26 E	10.34 ± 0.67 F	1.38 ± 0.11 D
TS17B15K3.0-122	609.90 ± 0.44 E	56.63 ± 0.14 A	1012 ± 2 H	4.99 ± 0.32 D	12.39 ± 1.00 D	1.47 ± 0.11 C

^1^ Each value is expressed as mean ± standard deviation (*n* = 6 for hardness and staling rate, *n* = 3 for other qualities). Means with different capital letters within a column differ significantly (*p* < 0.05).

## Data Availability

The original contributions presented in the study are included in the article. Further inquiries can be directed to the corresponding author.

## Abbreviations

The following abbreviations are used in this manuscript:

TS17	Taichung sen 17
B	Indica black rice
K	Konjac glucomannan
